# Identifying clinical features associated with electroconvulsive therapy response in adolescents with major depressive disorder using machine learning

**DOI:** 10.3389/fpsyt.2026.1824658

**Published:** 2026-05-07

**Authors:** Bingyang Zha, Linjie Li, Jinglan He, Jun Cao, Su Hong, Li Kuang

**Affiliations:** 1 Department of Psychiatry, The First Affiliated Hospital of Chongqing Medical University, Chongqing, China; 2 Mental Health Center, The First Affiliated Hospital of Chongqing Medical University, Chongqing, China; 3 Department of Urology, The First Affiliated Hospital of Chongqing Medical University, Chongqing, China

**Keywords:** adolescent major depressive disorder, electroconvulsive therapy, machine learning, risk stratification, treatment response

## Abstract

**Background:**

Electroconvulsive therapy (ECT) is an effective treatment for adolescent major depressive disorder (MDD), but its efficacy varies. This study utilized machine learning (ML) to identify baseline clinical factors associated with poor ECT response.

**Methods:**

We retrospectively enrolled 503 adolescent MDD patients. A poor response was defined as a <50% reduction on the Hamilton Depression Scale (HAMD-24). The optimal ML algorithm (Random Forest, RF) was selected from nine candidates and then simplified using recursive feature elimination (RFE) and interpreted via Shapley Additive Explanations (SHAP).

**Results:**

A simplified model using two baseline features—the neutrophil-to-platelet ratio (NPR) and pre-treatment HAMD score—achieved an AUC of 0.731 on the testing set, comparable to the full-feature model (AUC: 0.751). SHAP analysis revealed that a lower baseline NPR and a lower pre-treatment HAMD score were associated with a poor response. Furthermore, retrospective statistical comparisons revealed that patients in the poor response group completed significantly fewer ECT sessions than those in the good response group.

**Conclusions:**

We developed a concise explanatory model demonstrating that routine clinical data available at admission (blood NPR and HAMD score) can effectively stratify the risk of poor ECT efficacy. Crucially, identifying these high-risk patients early empowers clinicians to implement targeted management, ensuring they complete a full and adequate course of ECT to maximize therapeutic benefits and prevent premature termination.

## 1 Introduction

The incidence of Major Depressive Disorder (MDD) in adolescents is increasing annually, representing a severe public health challenge that significantly impairs academic performance, social functioning, and elevates suicide risk (1–4). While pharmacotherapy and psychotherapy are first-line treatments, a significant proportion of patients exhibit a poor response, leading to a treatment-resistant course (5). For adolescents with severe, treatment-resistant MDD, psychotic symptoms, or intense suicidal ideation, Electroconvulsive Therapy (ECT) can rapidly alleviate symptoms and is a critical intervention (6–9). However, the efficacy of ECT is not universal and varies considerably among individuals. Clinical studies indicate that approximately 20–30% of patients respond poorly to ECT, and this heterogeneity poses a significant clinical challenge (10). Therefore, accurately identifying individuals at high risk for a poor response before or early in the treatment course is crucial for optimizing resource allocation, managing patient expectations, and reducing unnecessary treatment-related risks (11).

Previous research attempting to predict ECT outcomes has often faced limitations. Many studies have focused on single-domain predictors, such as individual inflammatory markers like C-reactive protein or interleukin-6, which may be insufficient to reflect the body’s complex immune status (12, 13). In contrast, composite inflammatory markers derived from routine blood tests (e.g., NLR, PLR, NPR) offer a more comprehensive and economical alternative (14, 15). Furthermore, uncovering meaningful patterns from such multidimensional data requires advanced analytical methods beyond traditional statistics. While machine learning (ML) has emerged as a novel approach to address this need (16–18), early applications sometimes relied on black-box models, which limit clinical interpretability and hinder a deeper understanding of the factors driving treatment response.

This study aims to address these gaps by using ML methods to integrate multidimensional baseline data, including composite inflammatory indices and clinical scales. Our goal is to construct a robust early-warning predictive model that aligns with clinical reality by focusing on pre-treatment predictors. Additionally, we analyze the number of completed ECT sessions as an observational clinical factor to discuss how baseline risk and the adequacy of the treatment process jointly impact the final efficacy.

## 2 Materials and methods

### 2.1 Study participants

**Figure 1:**
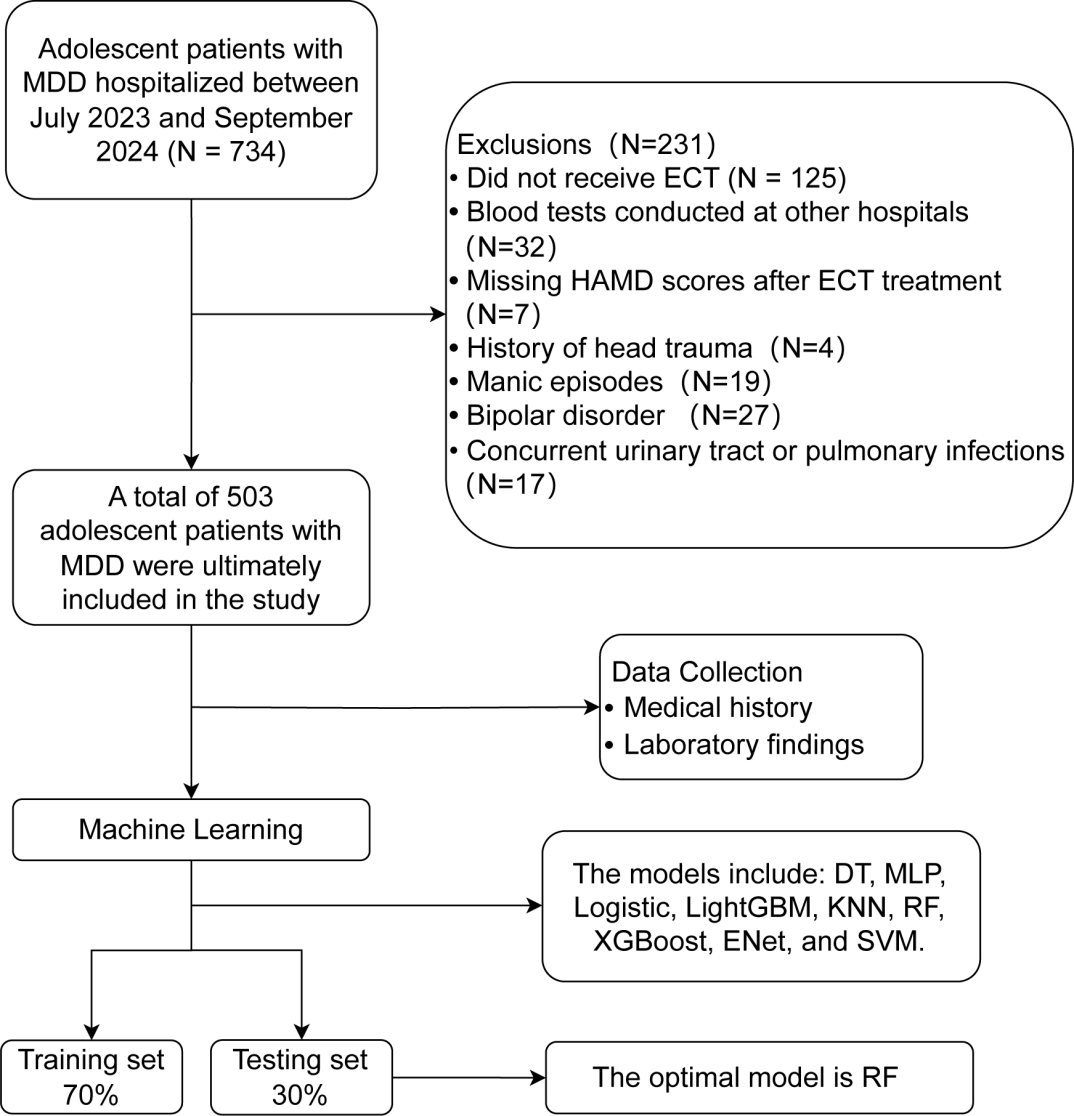
Flowchart of patient screening for the study. This study initially screened 734 adolescent MDD patients. According to the pre-set inclusion and exclusion criteria, 503 patients were ultimately included in the analysis and were randomly divided into a training set and a validation set. Nine machine learning algorithms were trained on the training set to build models, and model performance was evaluated on the validation set. Ultimately, RF was selected as the final model.

This retrospective study analyzed the medical records of adolescent MDD patients who were hospitalized and received ECT at the Department of Psychiatry, The First Affiliated Hospital of Chongqing Medical University, between July 2023 and September 2024. A total of 734 patient records were initially screened. The inclusion criteria were: (1) age between 13 and 18 years; (2) a clear diagnosis of MDD according to the Diagnostic and Statistical Manual of Mental Disorders, Fifth Edition (DSM-5); (3) non-response to at least two adequate trials of different antidepressants; (4) HAMD-24 score ≥ 35; (5) received ECT treatment; and (6) completed a routine blood test within 24 hours of admission. The exclusion criteria were: (1) comorbid manic episodes or bipolar disorder; (2) history of organic brain disease or severe head trauma; (3) comorbid severe or unstable physical illnesses; (4) presence of autoimmune diseases, active infections, or use of anti-inflammatory or immunosuppressive therapy within the past two weeks; (5) history of substance dependence or abuse; (6) missing values in records (specifically, 32 patients were excluded as their routine blood tests were conducted at other hospitals, making core inflammatory features untraceable, as shown in **Figure 1**); and (7) intellectual disability (Intelligence Quotient < 70). Based on the inclusion and exclusion criteria, a final sample of 503 adolescent MDD patients was enrolled in the analysis (**Figure 1**). Importantly, all included patients had been followed until their clinical ECT regimen was either fully completed or officially terminated by their attending physicians prior to our data extraction cutoff (September 2024). Therefore, the number of completed ECT sessions reflects the actual total sessions received by the patient during that hospitalization, rather than an artificial truncation due to the study timeline. Because our core features rely on complete blood counts, omitting patients without these specific records was deemed necessary to maintain biological validity rather than using mathematical imputation on entirely blank biological matrices.

### 2.2 Study variables

We collected the following clinical data from the electronic medical record system:

1. Demographic and Clinical Characteristics: Age, gender, height, weight, body mass index (BMI), disease duration, history of suicidal behavior, psychotic symptoms, sleep disturbance, loss of appetite, history of ECT, number of previous ECT sessions, smoking status, and drinking status. Furthermore, patients were considered overweight if they had a BMI ≥ 25 kg/m ².
2. Medication Usage: Various psychiatric medications used during treatment, including antidepressants, antipsychotics, and anxiolytics.
3. Blood Inflammatory Markers: Results from routine blood tests within 24 hours of admission, including white blood cell count (WBC), hemoglobin, platelets, and the absolute counts and percentages of neutrophils, lymphocytes, and monocytes. Several composite inflammatory indices were calculated, such as the neutrophil-to-lymphocyte ratio (NLR), neutrophil-to-platelet ratio (NPR), and platelet-to-lymphocyte ratio (PLR). We selected these indices based on the following considerations: first, they are derived from routine, low-cost complete blood count tests, making them easy to popularize; second, the inflammatory response is confirmed to be related to the pathophysiology of depression and treatment response (13, 19); finally, compared to individual cell counts, composite indices such as NPR and NLR may more stably and comprehensively reflect the balance between neutrophils, lymphocytes, and platelets, thereby potentially providing a more accurate depiction of the systemic inflammatory state.
4. Questionnaire Scores and ECT Parameters: Pre- and post-treatment scores on the HAMD-24 and Hamilton Anxiety Scale (HAMA) (20, 21), as well as the number of completed ECT sessions.

### 2.3 Electroconvulsive therapy procedure

As this was a retrospective medical chart review, the ECT procedures described below reflect the standard clinical protocols administered to the patients during their hospitalization, rather than a prospective study-specific intervention. All patients received modified ECT. Before treatment, after verifying patient information, intravenous access was established, and monitoring devices for electrocardiogram, blood pressure, oxygen saturation, and electroencephalogram were connected. ECT was administered using a Thymatron DGx system (Somatics, LLC, USA) with bilateral temporal electrode placement and a brief-pulse stimulus. The initial charge was estimated using the *age × 0.7* formula, and the stimulus energy for subsequent treatments was adjusted based on the quality of the previous seizure (primarily referencing the seizure duration on EEG). If the seizure duration was less than 25 seconds, the energy was increased by 5% in the next session. Anesthesia and muscle relaxation were achieved with propofol (1.5–2 mg/kg) and succinylcholine (0.5–1 mg/kg), respectively. Atropine was used as needed to regulate heart rate.

### 2.4 Outcome measurement

The primary outcome of this study was the treatment response at the end of the ECT course. We defined treatment response as follows: the Poor Response Group had a HAMD-24 reduction rate of < 50%, and the Good Response Group had a HAMD-24 reduction rate of ≥ 50%. The HAMD-24 reduction rate was calculated as: [(pre-treatment total score - post-treatment total score)/pre-treatment total score] × 100%. In this study, poor response was set as the positive event for model construction and evaluation.

### 2.5 Machine learning algorithms

Given that no significant differences were observed in the types of medications used between the poor response group and the good response group, and considering the complex and multifaceted interactions of pharmacotherapy with ECT efficacy, all baseline variables, with the exception of medication data and treatment process variables (ECT sessions), were included in the subsequent machine learning analysis.

#### 2.5.1 Data preprocessing

Continuous variables were standardized using Z-scores, and categorical variables were one-hot encoded. To address potential class imbalance, we evaluated model performance both with and without the Synthetic Minority Over-sampling Technique (SMOTE). To maintain the model’s interpretability and ensure that the feature importance directly reflected the original clinical variables, no other complex feature engineering techniques were applied beyond these fundamental preprocessing steps.

#### 2.5.2 Dataset splitting

The data from 503 patients were randomly split into a training set and a testing set at a 7:3 ratio.

#### 2.5.3 Model building and optimization

Nine machine learning models were built on the training set: Decision Tree (DT), Multi-Layer Perceptron (MLP), Logistic Regression (Logistic), Light Gradient Boosting Machine (LightGBM), K-Nearest Neighbors (KNN), Random Forest (RF), eXtreme Gradient Boosting (XGBoost), Elastic Net (ENet), and Support Vector Machine (SVM). These algorithms were selected to comprehensively evaluate linear, distance-based, tree-based, and neural network approaches on our clinical dataset. For each algorithm, we performed an automated hyperparameter grid search and optimization using five-fold cross-validation.

#### 2.5.4 Model evaluation

The performance of the final models was evaluated on the testing set using metrics including Accuracy, Sensitivity, Specificity, Balanced Accuracy, and AUC.

#### 2.5.5 Feature selection and model simplification

To enhance clinical utility, we performed feature selection. First, we assessed the global importance of all features using SHAP analysis. Subsequently, we employed the RFE method to systematically reduce the number of features by repeatedly building an RF model and eliminating the least important feature from the previous iteration. By observing the model’s AUC curve on the testing set as the number of features decreased, we determined the optimal feature subset that could maintain high performance while maximizing model simplification.

#### 2.5.6 Model interpretation

The SHAP algorithm was used to interpret the final simplified model, quantifying the contribution of each key feature to the model’s output, thereby revealing the internal logic of how each factor is associated with the treatment outcome.

### 2.6 Data processing and statistical analysis

Data analysis was performed using R software (version 4.3.2) and SPSS software (version 25.0). The normality of quantitative variables was first tested using the Shapiro-Wilk test. Normally distributed variables were described as mean ± standard deviation (Mean ± SD), and comparisons between groups were made using independent samples t-tests. Non-normally distributed variables were expressed as median and interquartile range [Median (Q1, Q3)], and comparisons were made using the Mann-Whitney U test. Categorical variables were presented as frequency and percentage [n(%)], and comparisons were made using the Pearson chi-squared test or Fisher’s exact test. All statistical tests were two-sided, with the significance level set at α = 0.05.

## 3 Results

### 3.1 Baseline characteristics

A total of 503 adolescent MDD patients were included in this study and were divided into a poor response group (n=127, 25.35%) and a good response group (n=376, 74.75%) based on their ECT treatment outcomes. As shown in **Tables 1**, **2**, no statistically significant differences were observed between the two groups in demographic and clinical characteristics such as age, gender, BMI, disease duration, history of psychotic symptoms, history of suicidal behavior, or in the use of various medications (all P > 0.05). Regarding the number of previous ECT sessions, the mean ± standard deviation was 0.86 ± 3.15 for the poor response group, and 1.01 ± 3.05 for the good response group, showing no statistically significant difference. Several other variables, including the Neutrophil-to-Lymphocyte Ratio (NLR; P = 0.082), monocyte percentage (P = 0.088), and the use of fluvoxamine maleate (P = 0.094), also showed no statistically significant differences (**Tables 2**, **3**).

**Table 1:** Demographic and Clinical Characteristics.

Variables	Treatment Response (n = 503)		χ²/Z value^2^	P value^2^
	Poor^1^ (n = 127)	Good^1^ (n = 376)		
Age (years)	15.00 (13.50,16.00)	15.00 (13.00,16.00)	-0.416	0.678
Female	103 (81.10%)	289 (76.86%)	0.993	0.319
Height (m)	1.63 (1.57,1.67)	1.62 (1.58,1.67)	-0.127	0.899
Weight (kg)	53.00 (45.00,62.50)	52.00 (47.50,61.25)	-0.204	0.839
BMI (kg/m²)	20.00 (17.96,23.53)	19.97 (18.32,22.80)	-0.082	0.935
Disease Duration (years)	2.00 (1.00,3.00)	1.00 (1.00,3.00)	-1.060	0.290
History of Suicidal Behavior	111 (87.40%)	343 (91.22%)	1.580	0.209
Psychotic Symptoms	91 (71.65%)	253 (67.29%)	0.837	0.360
Sleep Disturbance	116 (91.34%)	352 (93.62%)	0.761	0.383
Loss of Appetite	87 (68.50%)	274 (72.87%)	0.894	0.344
History of ECT	11 (8.66%)	43 (11.44%)	0.763	0.382
Number of Previous ECT Sessions	0.86 ± 3.15	1.01 ± 3.05	-0.865	0.387
Smoking Status	10 (7.87%)	23 (6.12%)	0.478	0.489
Drinking Status	7 (5.51%)	19 (5.05%)	0.041	0.840

^1^n (%); Mean ± SD; Median (Q1,Q3).

^2^Pearson's Chi-squared test; Fisher's exact test; Wilcoxon rank sum test.

**Table 2:** Medication Usage.

Variables	Treatment Response (n = 503)		χ²/Z value^2^	P value^2^
	Poor^1^ (n = 127)	Good^1^ (n = 376)		
Sertraline	56 (44.09%)	153 (40.69%)	0.453	0.501
Fluoxetine	27 (21.26%)	89 (23.67%)	0.311	0.577
Fluvoxamine Maleate	9 (7.09%)	47 (12.50%)	2.810	0.094
Citalopram	6 (4.72%)	22 (5.85%)	0.229	0.632
Duloxetine	3 (2.36%)	18 (4.79%)	1.400	0.237
Venlafaxine	6 (4.72%)	8 (2.13%)	2.370	0.214
Tandospirone	50 (39.37%)	121 (32.18%)	2.190	0.139
Bupropion	12 (9.45%)	24 (6.38%)	1.340	0.247
Quetiapine	63 (49.61%)	185 (49.20%)	0.006	0.937
Olanzapine	36 (28.35%)	106 (28.19%)	0.001	0.973
Aripiprazole	13 (10.24%)	60 (15.96%)	2.500	0.114
Risperidone	3 (2.36%)	8 (2.13%)	0.024	1.000
Oxazepam	20 (15.75%)	49 (13.03%)	0.592	0.442
Alprazolam	17 (13.39%)	38 (10.11%)	1.050	0.306
Clonazepam	9 (7.09%)	14 (3.72%)	2.460	0.117
Zopiclone	6 (4.72%)	10 (2.66%)	1.310	0.382

^1^n (%); Mean ± SD; Median (Q1,Q3).

^2^Pearson's Chi-squared test; Fisher's exact test; Wilcoxon rank sum test.

**Table 3:** Blood inflammatory markers.

Variables	Treatment Response (n = 503)		χ²/Z value^2^	P value^2^
	Poor^1^ (n = 127)	Good^1^ (n = 376)		
WBC (10^9^/L)	5.85 (4.87,7.04)	6.20 (5.33,7.30)	-2.320	0.021*
Hemoglobin (10^9^/L)	126.00 (120.00,135.00)	129.00 (121.00,138.00)	-1.360	0.174
Platelets (10^9^/L)	265.00 (222.00,304.50)	255.00 (220.00,304.00)	-1.060	0.290
Neutrophils (10^9^/L)	2.80 (2.32,3.40)	3.00 (2.46,3.88)	-2.590	0.010*
Lymphocytes (10^9^/L)	2.37 (1.95,2.80)	2.41 (1.97,2.84)	-0.273	0.785
Monocytes (10^9^/L)	0.46 (0.38,0.56)	0.46 (0.38,0.57)	-0.464	0.642
Neutrophil Percentage (%)	0.48 (0.44,0.53)	0.50 (0.44,0.56)	-1.970	0.049*
Lymphocyte Percentage (%)	0.40±0.07	0.39±0.09	2.210	0.028*
Monocyte Percentage (%)	0.08 (0.06,0.09)	0.07 (0.06,0.09)	-1.700	0.088
NLR	1.19 (0.97,1.50)	1.30 (0.96,1.68)	-1.740	0.082
NPR	0.01 (0.01,0.01)	0.01 (0.01,0.02)	-3.150	0.002**
PLR	106.01 (91.07,138.30)	107.45 (88.93,133.07)	-0.966	0.334
LMR	5.11 (4.09,6.12)	5.09 (4.13,6.56)	-0.223	0.823
MLR	0.20 (0.16,0.24)	0.20 (0.15,0.24)	-0.223	0.823
SIRI	0.55 (0.40,0.75)	0.60 (0.40,0.85)	-1.600	0.108
SII	301.01 (247.08,412.61)	332.27 (236.96,466.85)	-1.060	0.287
AISI	147.71 (98.11,207.22)	147.46 (99.81,239.76)	-1.060	0.288

^1^ n (%); Mean ± SD; Median (Q1,Q3).

^2^ Pearson's Chi-squared test; Fisher's exact test; Wilcoxon rank sum test.

Significance markers : **P < 0.01, *P < 0.05.

However, significant differences were observed in blood inflammatory markers and clinical scale scores. According to **Table 3**, compared to the good response group, patients in the poor response group had a lower NPR at admission [median (IQR): 0.01 (0.01, 0.01) vs. 0.01 (0.01, 0.02), P = 0.002]. This statistically significant difference, despite identical medians, was primarily driven by the distribution’s upper tail, with the good-response group showing a higher upper quartile (0.02) compared to the poor-response group (0.01). Additionally, the poor response group had lower white blood cell (WBC) counts, absolute neutrophil counts, and neutrophil percentages (all P < 0.05), while their lymphocyte percentage was higher (P = 0.028). According to **Table 4**, patients in the poor response group received fewer ECT sessions than those in the good response group [median (IQR): 7.00 (6.00, 9.00) vs. 8.00 (6.00, 9.00), P = 0.034]. Concurrently, the poor response group had lower pre-treatment HAMD scores (33.42 ± 8.80 vs. 35.78 ± 9.25, P = 0.011) and HAMA scores [median (IQR): 23.00 (19.00, 27.00) vs. 24.00 (20.00, 30.00), P = 0.046] compared to the good response group. After the treatment course, the HAMA and HAMD scores were higher in the poor response group (both P<0.001). These variables with significant differences constituted important candidate features for subsequent model building.

**Table 4:** Questionnaire scores and number of ECT sessions.

Variables	Treatment Response (n = 503)		χ²/Z value^2^	P value^2^
	Poor^1^ (n = 127)	Good^1^ (n = 376)		
Number of ECT Sessions	7.00 (6.00,9.00)	8.00 (6.00,9.00)	-2.120	0.034*
HAMA Score Before ECT	23.00 (19.00,27.00)	24.00 (20.00,30.00)	-2.000	0.046*
HAMA Score After ECT	14.00 (11.00,18.00)	8.00 (5.00,11.00)	-11.300	P<0.001***
HAMD Score Before ECT	33.42±8.80	35.78±9.25	-2.580	0.011*
HAMD Score After ECT	21.00 (17.00,25.00)	10.00 (7.00,14.00)	-13.700	P<0.001***

^1^ n (%); Mean ± SD; Median (Q1,Q3).

^2^ Pearson's Chi-squared test; Fisher's exact test; Wilcoxon rank sum test.

Significance markers : ***P < 0.001, **P < 0.01, *P < 0.05.

### 3.2 Optimal algorithm selection

**Figure 2:**
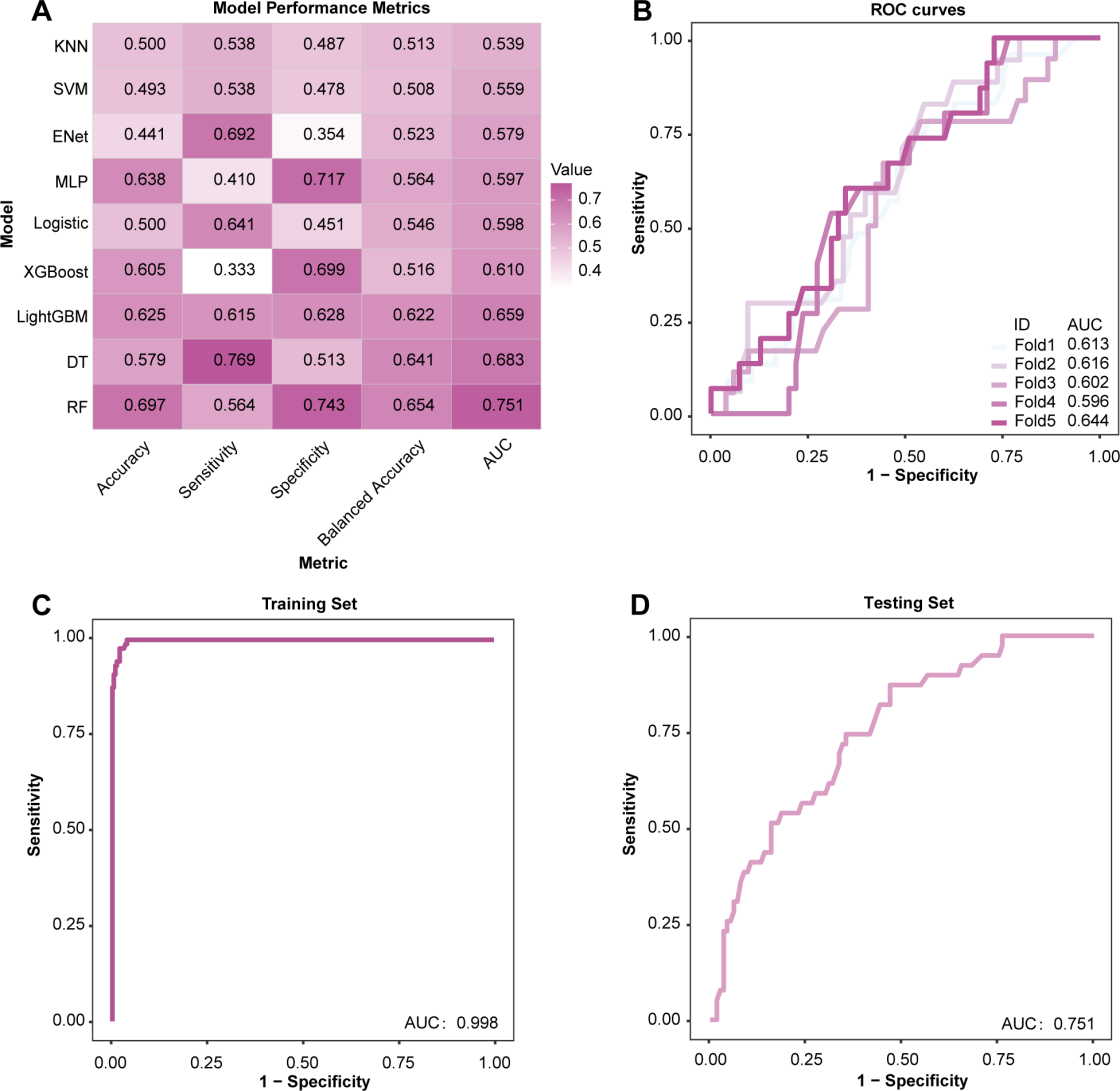
Performance comparison of multiple machine learning models. **(A)** Heatmap of performance metrics for nine machine learning models on the testing set. **(B)** Receiver operating characteristic (ROC) curves for the RF model during five-fold cross-validation in the training set. **(C, D)** ROC curves for the full-feature RF model on the training and testing sets.

To select the algorithm with the best identification performance, we trained nine machine learning models on the training set and validated them on the testing set (hyperparameters for each model are in **Supplementary Table 1**). We evaluated the nine models on both SMOTE-augmented data (**Supplementary Figure 1**) and the original unresampled data (**Figure 2A**). Comparing the performance metrics, we found that applying SMOTE did not yield a distinct advantage and could potentially distort the real-world clinical distribution of our data. Therefore, we ultimately chose to proceed with the unresampled approach. Overall, the RF model demonstrated the most balanced and optimal performance across all metrics, particularly achieving the highest value of 0.751 on the key discrimination metric, AUC. **Figure 2B** shows its ROC curves during five-fold cross-validation within the training set, demonstrating the stability of the model training process. In contrast, some other models performed poorly.

**Figures 2C, D** show the ROC curves of the full-feature RF model on the training and testing sets, respectively. The model achieved an AUC of 0.998 (95% CI [0.996-1.000]) on the training set and an AUC of 0.751 (95% CI [0.666-0.836]) on the testing set. Although there is a gap between the two, it is within an acceptable range, indicating that the model has good generalization ability and no significant overfitting was observed. Therefore, we selected RF as the base model for subsequent feature simplification and interpretability analysis.

### 3.3 Model simplification and evaluation

**Figure 3:**
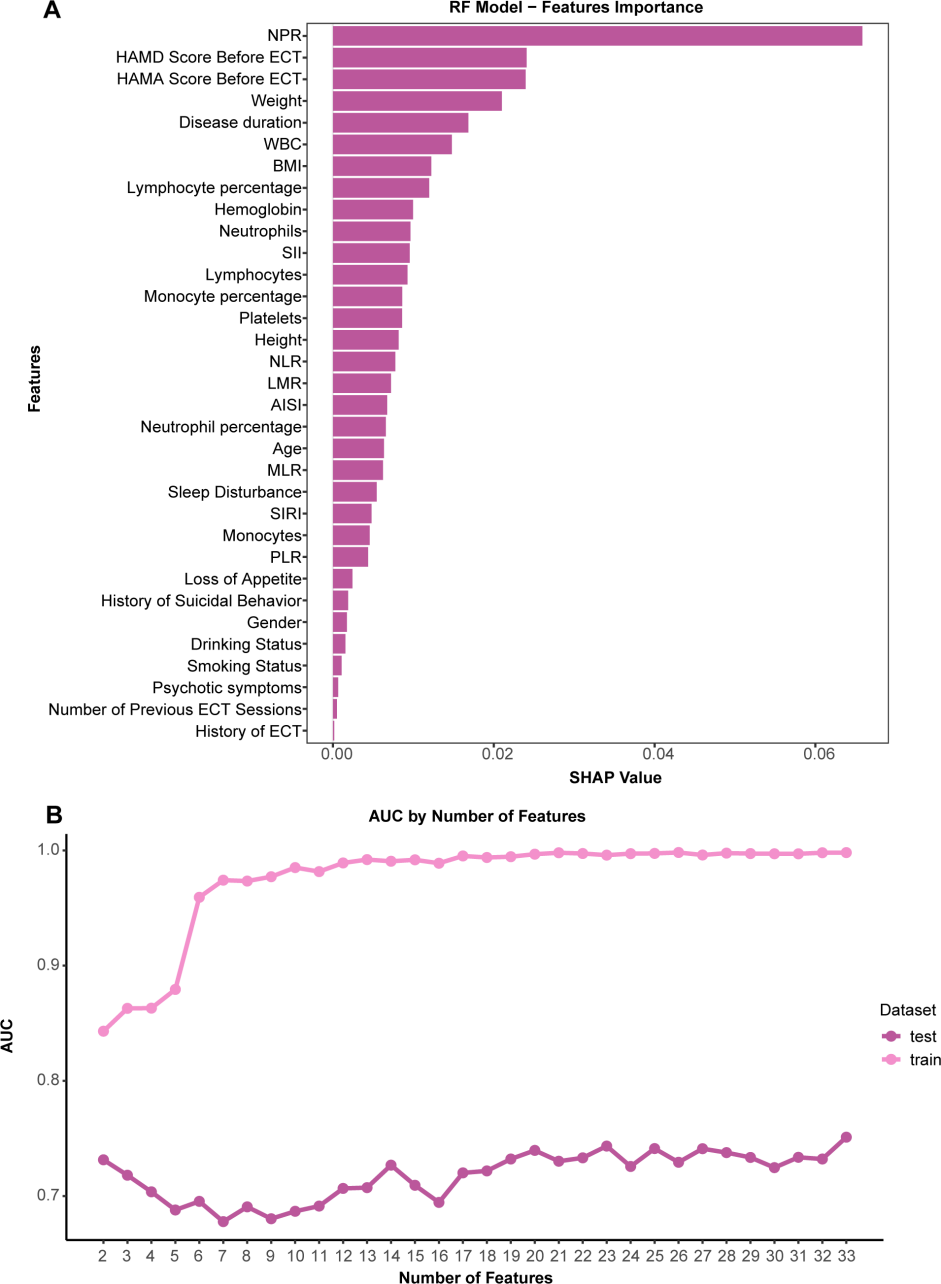
Model feature simplification. **(A)** Global feature importance ranking plot based on SHAP values, showing the mean absolute impact of each feature on the identification results in the RF model. **(B)** Curve showing the change in model performance with the number of features when using the RFE method.

Although the RF model including all variables showed good performance, collecting dozens of variables in clinical practice increases application difficulty and cost. To enhance the model’s simplicity and clinical usability, we performed feature selection. **Figure 3A** shows the global importance ranking of all features in the RF model based on SHAP values, where NPR and pre-treatment HAMD score demonstrated strong overall influence on the model’s identification.

Next, we used the RFE method to explore the optimal number of features. As shown in **Figure 3B**, as the number of features was gradually reduced from all variables, the AUC on the testing set fluctuated between 0.678 and 0.751. When the number of features was reduced to two, the performance did not show a significant decline compared to the full-feature model. These two core features were the top two variables in the importance ranking: NPR and pre-treatment HAMD score.

**Figure 4:**
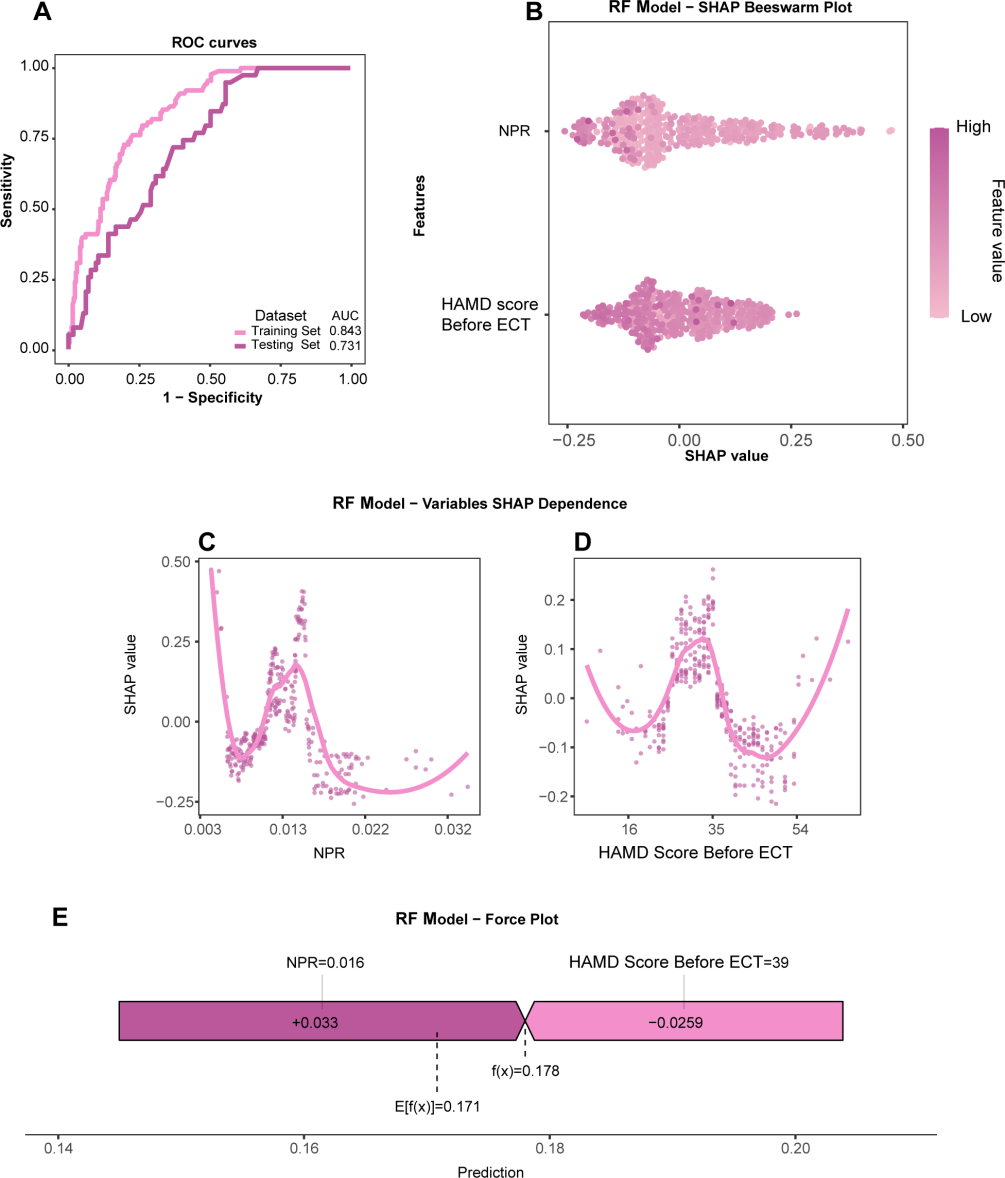
SHAP interpretability analysis of the model. **(A)** ROC curves for the three-feature RF model on the training and testing sets. **(B)** SHAP beeswarm plot for the three features, showing the impact of each feature’s value and distribution on the model’s output. **(C, D)** SHAP dependence plots for each feature, showing the relationship between each feature’s value and its corresponding SHAP value. **(E)** SHAP force plot for a single sample prediction, illustrating how the feature values push the prediction from the baseline value to the final output value.

We retrained a simplified RF model using these two features (the model’s hyperparameters are in **Supplementary Table 2**). **Figure 4A** shows the ROC curves of this simplified model on the training and testing sets. The results showed that the simplified model achieved an AUC of 0.731 (95% CI [0.649-0.814]) on the testing set, which is very close to the model using all features. This result indicates that the simplified model maintained a good discriminative ability while reducing the data collection burden for clinical application, thus achieving a favorable balance between performance and practicality for potential generalization.

To further evaluate the stability and generalizability of the simplified RF model, we conducted a stratified analysis to examine its performance in different clinical subgroups (**Table 5**). The results showed that the model demonstrated consistent and robust identification performance in patient subgroups of different genders (male AUC: 0.830 vs. female AUC: 0.804, P = 0.603), overweight status (yes AUC: 0.820 vs. no AUC: 0.804, P = 0.77), and presence of psychotic symptoms (present AUC: 0.832 vs. absent AUC: 0.750, P = 0.076). The results of the DeLong test indicated that there were no statistically significant differences in the AUC values of the model among these subgroups (all P > 0.05). While these preliminary subgroup analyses show no statistical difference and suggest a potential for generalization, the findings should be interpreted with caution, particularly in smaller subgroups like the overweight group (n=74) where statistical power is limited. Therefore, the robustness of these results needs to be confirmed in larger, external datasets.

**Table 5:** RF model performance in subgroups.

Variables	Sample size	Accuracy	Sensitivity	Specificity	Balanced Accuracy	AUC(95%CI)	P value^1^
Gender							0.603
Male	111	0.792	0.542	0.862	0.702	0.830 (0.743-0.917)	
Female	392	0.765	0.505	0.858	0.682	0.804 (0.760-0.848)	
Overweight							0.770
Yes	74	0.743	0.591	0.808	0.699	0.820 (0.724-0.916)	
No	429	0.776	0.495	0.867	0.681	0.804 (0.760-0.847)	
Psychotic symptoms							0.076
Present	344	0.794	0.560	0.878	0.719	0.832 (0.787-0.877)	
Absent	159	0.723	0.389	0.821	0.605	0.750 (0.672-0.828)	

^1^ DeLong's test.

### 3.4 Model interpretation and application

To understand how the simplified two-feature model makes its identifications, we employed the SHAP method for interpretability analysis. Specifically, a lower baseline NPR and a lower baseline HAMD score are the two core factors closely associated with a poor ECT treatment response (**Figure 4B**). The SHAP dependence plots further reveal the relationship between each feature and the identification result (**Figures 4C, D**).

The SHAP force plot illustrates the model’s identification process for a specific sample (**Figure 4E**). The population baseline prediction value for this sample was 0.171. For this particular sample, its low NPR value (0.016) was the main driver pushing the model to identify it as a poor response, while its high HAMD score (39) acted as a protective factor, reducing the probability of being identified as having a poor response. Under the combined effect of these factors, the final prediction value given by the model was 0.178 which is higher than the baseline value, thus identifying this patient as having a poor response.

## 4 Discussion

This study applied machine learning algorithms to explore multidimensional factors associated with ECT response in adolescent MDD patients. Our core contribution is the development and validation of a simplified RF model that requires only two routine clinical variables—NPR and baseline HAMD score—to effectively identify patients with a poor treatment response. Furthermore, the stratified analysis indicated that the model’s performance remained stable across patients of different genders, weight statuses, and presence of psychotic symptoms, highlighting its reliable generalization ability.

The machine learning model’s ability to identify subtle predictors that traditional methods might overlook allowed it to capture the association between a lower NPR and poor treatment response, despite the statistically small absolute difference. This finding may, in turn, reveal a deeper mechanism of action for ECT. ECT itself has been shown to have anti-inflammatory and immunomodulatory effects (19, 22). For patients with a pre-existing low-grade inflammatory state (potentially reflected by a higher NPR), ECT might exert its antidepressant effects more effectively by correcting their immune system imbalance. Indeed, this view is supported by other studies; a meta-analysis indicated that patients with higher baseline inflammation levels are more likely to benefit from ECT, with higher baseline C-reactive protein and interleukin-6 levels being significantly associated with a greater reduction in depressive symptoms post-ECT, making this baseline inflammatory state a potential good efficacy indicator (13, 23). Conversely, for patients without high baseline inflammation, the pathophysiology of their depression may rely more on non-immune pathways, making them less sensitive to the immunomodulatory effects of ECT. Of course, this is only an inference based on the current data. The exact role of NPR as a biomarker for ECT efficacy and its underlying mechanisms urgently require further clarification through future prospective studies and molecular biology experiments. Furthermore, NPR’s significant association with the outcome may be explained by its ability to reflect the crosstalk between neutrophils (key inflammatory effectors) and platelets (immune modulators). Their interaction can form pro-inflammatory neutrophil-platelet aggregates, making NPR a more comprehensive measure of the underlying immune state than single-cell counts (24, 25).

Interestingly, our model identified that a lower baseline HAMD score is predictive of a poor response. While this indicates that patients with milder baseline symptoms are less likely to be classified as responders, we acknowledge that this may partially be driven by a statistical *floor effect*. Since response is defined as a ≥50% reduction in HAMD scores, patients with lower initial scores have a narrower numerical margin to achieve this proportional threshold compared to severely ill patients. Nonetheless, this aligns with the clinical consensus that ECT yields the most profound relative improvements in the most severe cases (26, 27).

While our ML model stratifies baseline risk, predicting outcomes at admission is only the first step in clinical management. Our statistical analysis highlights another crucial dimension: the treatment process itself. Specifically, patients in the poor response group completed significantly fewer ECT sessions compared to responders. In clinical practice, adolescents may prematurely terminate ECT due to poor tolerance of side effects (e.g., transient memory impairment, severe headache, or nausea), stigma-related anxiety, or parental withdrawal of consent. This finding serves as a powerful warning signal in clinical practice. It reminds clinicians that a complete course of ECT typically requires 8–12 sessions to achieve optimal and stable effects (28, 29). Throughout the treatment process, it is essential to be highly vigilant about factors that could lead to premature discontinuation (such as early adverse reactions, patient expectation management, financial pressures, etc.). Even if a patient is identified as high-risk at baseline, clinicians must emphasize the importance of completing the recommended course of treatment to achieve final efficacy, thereby combating pseudo-treatment resistance caused merely by insufficient treatment. Additionally, although the history of previous ECT did not show a statistically significant difference between the two groups in our cohort, clinicians should recognize that a history of ECT might influence clinical outcomes by affecting a patient’s level of hopefulness and treatment expectations. This psychological dimension warrants further attention in future prospective studies.

The clinical value of this study lies in its simplicity and practicality. For newly admitted adolescent patients with MDD, clinicians can perform an initial risk stratification using routine blood test results and HAMD scores. The model particularly suggests that special attention should be paid to patients with low baseline HAMD scores and low peripheral blood NPR levels. Such patients may be a high-risk group for poor ECT response, and their final efficacy depends on treatment adherence and adequacy. Therefore, the treatment strategy for these patients should be more proactive: enhance dynamic monitoring of adverse reactions in the early stages of treatment and invest more effort in health education and supportive communication to maximize the likelihood of them completing a full course of treatment.

Although this study provides important clinical insights, its limitations must be acknowledged. First, as a single-center, retrospective study, the external validity of its results needs to be verified by future multi-center, prospective studies. Furthermore, the reasons for premature treatment termination were not collected. Although our statistical analysis showed a significant difference in completed sessions between the two outcome groups, the retrospective nature of this study makes it difficult to fully disentangle whether early non-response led to treatment discontinuation or if insufficient treatments caused the poor outcome. In future prospective designs, the planned number of treatments should be included as a baseline variable, while the actual number of completed sessions and reasons for discontinuation (e.g., adverse reactions, patient preference, economic factors) should be recorded in detail. Statistical methods such as survival analysis could be used to more precisely dissect the risk factors for treatment discontinuation. Second, this study only included inflammatory markers derived from routine blood tests. Future research should aim to integrate multi-modal data, such as more specific cytokine profiles (e.g., IL-6, TNF-α), neuroimaging data, and genomic data. This could not only lead to models that distinguish between different biological subtypes of MDD but also potentially discover biomarkers that are more sensitive to specific treatments like ECT. Finally, as an exploratory retrospective analysis, our study protocol was not preregistered.

## 5 Conclusions

This study, through machine learning methods, constructed and validated a concise baseline model aimed at predicting ECT efficacy in adolescent patients with major depressive disorder. The core contribution of the research is revealing that baseline bio-psychological states (NPR, HAMD score) can effectively stratify the risk of poor ECT efficacy upon admission. Furthermore, our clinical observations highlight that early prediction is only the first step; identifying high-risk patients must be coupled with rigorous treatment management. Ensuring that these specific patients complete a full and adequate course of treatment remains a core strategy for improving ultimate efficacy and preventing pseudo-treatment resistance. This study provides a new perspective and evidence to support the future design of more precise, individualized treatment pathways.

## Data Availability

The datasets presented in this study can be found in online repositories. The names of the repository/repositories and accession number(s) can be found in the article/**Supplementary Material**.
