# Supplementary figures and images for: Identifying clinical features associated with electroconvulsive therapy response in adolescents with major depressive disorder using machine learning
Source: Front Psychiatry. 2026 May 7;17:1824658. doi: 10.3389/fpsyt.2026.1824658 (PMC13190595; doi:10.3389/fpsyt.2026.1824658)

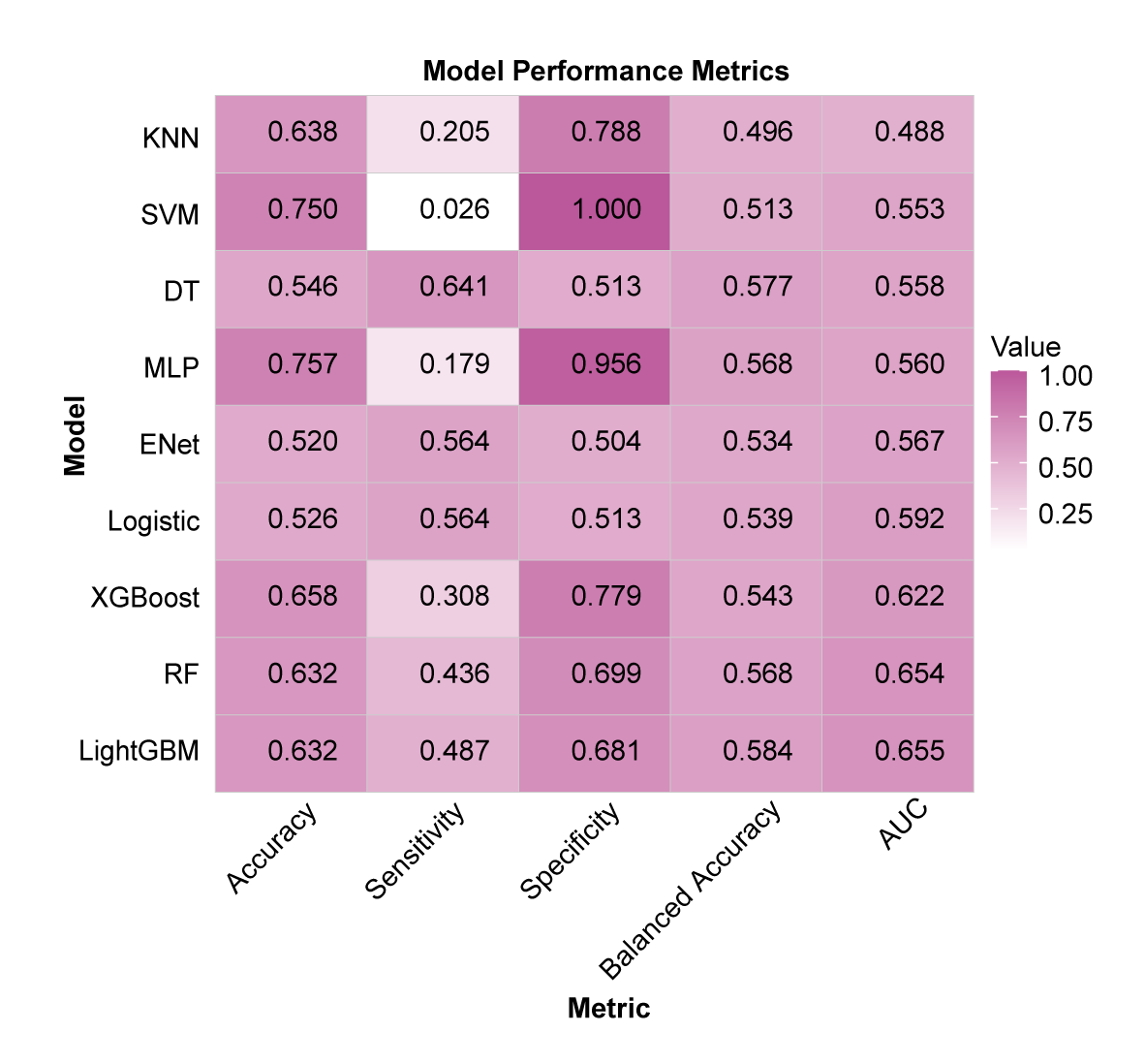

Supplement: Supplementary file 1 [file Image1.tif]
